# Leveraging Walnut Somatic Embryos as a Biomanufacturing Platform for Recombinant Proteins and Metabolites

**DOI:** 10.3390/biotech13040050

**Published:** 2024-11-15

**Authors:** Paulo A. Zaini, Katherine R. Haddad, Noah G. Feinberg, Yakir Ophir, Somen Nandi, Karen A. McDonald, Abhaya M. Dandekar

**Affiliations:** 1 Department of Plant Sciences, University of California, Davis, CA 95616, USA; pazaini@ucdavis.edu (P.A.Z.); ngfeinberg@ucdavis.edu (N.G.F.); 2 Department of Chemical Engineering, University of California, Davis, CA 95616, USA; khaddad@ucdavis.edu (K.R.H.); yo255@cornell.edu (Y.O.); snandi@ucdavis.edu (S.N.); kamcdonald@ucdavis.edu (K.A.M.); 3 Department of Microbiology and Immunology, Cornell University, Ithaca, NY 14850, USA

**Keywords:** walnut somatic embryos, tissue culture, biomanufacturing, diagnostic, vaccine, natural pigment

## Abstract

Biomanufacturing enables novel sources of compounds with constant demand, such as food coloring and preservatives, as well as new compounds with peak demand, such as diagnostics and vaccines. The COVID-19 pandemic has highlighted the need for alternative sources of research materials, thrusting research on diversification of biomanufacturing platforms. Here, we show initial results exploring the walnut somatic embryogenic system expressing the recombinant receptor binding domain (RBD) and ectodomain of the spike protein (Spike) from the SARS-CoV-2 virus. Stably transformed walnut embryo lines were selected and propagated in vitro. Both recombinant proteins were detected at 3–14 µg/g dry weight of tissue culture material. Although higher yields of recombinant protein have been obtained using more conventional biomanufacturing platforms, we also report on the production of the red pigment betanin in somatic embryos, reaching yields of 650 mg/g, even higher than red beet *Beta vulgaris*. This first iteration shows the potential of biomanufacturing using somatic walnut embryos that can now be further optimized for different applications sourcing specialized proteins and metabolites.

## 1. Introduction

The demand for diagnostic tests and vaccines surged during the COVID-19 pandemic, and despite the rapid increase in production capacity in 2020, critical supply shortages were widely experienced [1,2]. This prompted efforts to rapidly develop, manufacture, and distribute diagnostic tests and vaccines from new production centers and sources [3]. As a lesson from this pandemic, maintaining critical supply chains of both tests and vaccines can help reduce peaks in hospitalizations. These were among the greatest constraints that resulted in inadequate patient care and should be expected in future pandemics caused by severe respiratory viruses [4].

Both rapid tests and protein-based vaccines [5] contain recombinant proteins as a key component, using bacterial, fungal, and animal cells as their main biomanufacturing platform [6,7,8]. These employ massive bioreactors for cultivation and other sophisticated equipment to purify recombinant proteins, which runs counter to the goal of decentralizing and simplifying production. To this end, plants offer alternative platforms for biomanufacturing in the form of cell suspensions, hydroponic cultures, or whole-plant systems [9,10]. Some advantages of these systems include low production costs, scalability, lower risk of contamination with human pathogens, and proper eukaryotic protein folding and post-translational modifications [11,12,13]. Post-translational modifications can affect protein properties such as immunogenicity, binding, and stability, among other factors. These differences pose potential limitations to heterologous expression in plants, although efforts to modify the basal potential by genetic engineering [14] or in vitro glycan engineering have shown recent progress [15]. Furthermore, plants possess the necessary molecular machinery for diverse specialized metabolism, relevant to the production of small molecules [16]. One of the most developed plant systems for biomanufacturing is *Nicotiana benthamiana* leaf transient expression for both proteins and small molecules alike [17]. Rice cell suspension is also an established platform in a 40 L pilot-scale stirred tank bioreactor [18], as well as some other plant species [19].

Due to the declining demand for both vaccines and rapid tests from 2023 onwards, the new challenge of preserving manufacturing capacity to rapidly combat future waves of more severe variants has been posed. To this end of preparedness, the decentralization and diversification of local biomanufacturing capabilities have gained support from the National Institute of Standards and Technology (NIST) through the Bioindustrial Manufacturing and Design Ecosystem (BioMADE) [20] initiative that structured the Rapid Assistance (for) Coronavirus Economic Response (RACER) Grant Program. Our project includes the exploration of alternative antigen production platforms, for which we contributed our previous experience with walnut somatic embryo tissue cultures [21]. Walnut embryos have not been characterized in detail as a biomanufacturing platform but retain the expression and post-transcriptional modification capacity of other plants, with the additional advantage of being seed-like tissue and potentially edible with little or no additional processing requirements. Research has centered mostly on propagation [22], genomics [23,24], and gene editing [25] that could expand its development as a biomanufacturing platform.

Here, we report on the transgenic production of the recombinant proteins RBD (receptor binding domain, aa. 331-521) and SPIKE (full-length spike without transmembrane domain, aa. 14-1208) from the SARS-CoV-2 spike protein Spike_6P_D614G [26]. We also report on the production of the red pigment betanin, a valuable natural food additive for its antioxidant and coloring characteristics [27]. The vectors designed for the expression of recombinant proteins also included the RUBY scorable marker to hasten the identification and selection of successfully transformed individuals. The deployment of RUBY as a visual marker led to a surprising level of betanin accumulation due to the activity of the three enzymes (a cytochrome P450 encoded by CYP76AD1α, DODAα, and a glycosyl transferase) encoded in the RUBY synthetic gene not produced by wild-type cells. This indicates the system’s capacity for biomanufacturing of aromatic amino acid-derived small molecules.

## 2. Materials and Methods

### 2.1. Design and Construction of DNA Expression Vectors

**Figure 1:**
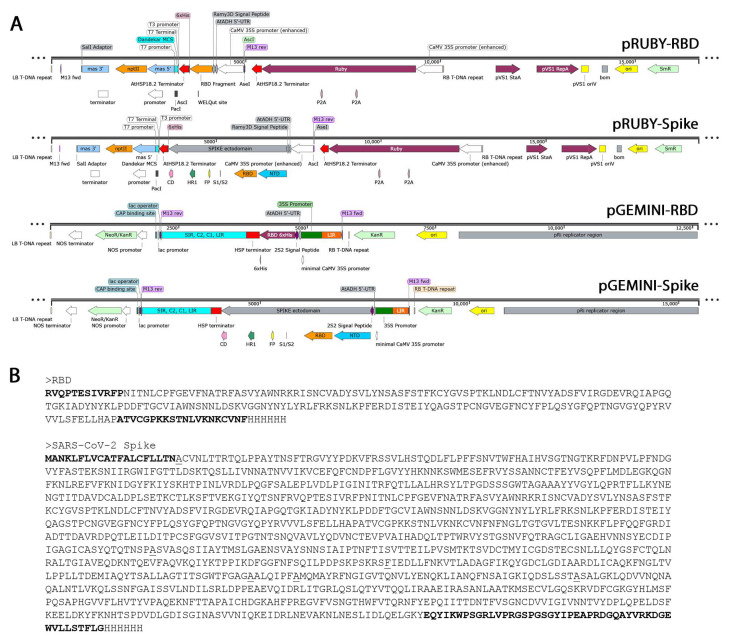
Diagram showing plant expression vectors used in this study. (**A**) Two sets of vectors were tested: one derived from Addgene vector 160908 expressing the red pigment betanin encoded by RUBY and the other derived from Takara vector pRI 201-AN with inserted geminiviral components for enhanced DNA replication [30,31]. In each vector type, both RBD (aa. 331-521 of full-length spike in pRUBY-RBD and aa. 319-541 in pGEMINI-RBD) and SPIKE ectodomain (aa. 36-1167 and 16-1209, respectively) of SARS-CoV-2 spike protein were encoded with a C-terminal HisTag. For RUBY vectors, variations in the expression cassette were tested for enhanced expression. This included no 5′-UTR sequence, SpeI restriction site, or the 5′-UTR present in the Gemini vector (from the *Arabidopsis thaliana* alcohol dehydrogenase ADH). (**B**) Sequences of recombinant proteins are shown for RBD and SPIKE. The sequences present only in pGEMINI are shown in bold, and the sites underlined are substituted with prolines in the SPIKE protein expressed in pRUBY.

Spike_6P_D614G (GenBank QHD43416.1 with modifications described in [26]) was the base for the design, codon-optimized to walnut using an online tool (Twist Bioscience, San Francisco, CA, USA), and synthesized by GenScript (Piscataway, NJ, USA), containing C-terminal 6xHisTag and AseI and PacI restriction sites at 5′ and 3′ of the synthetic DNA. The fragment was inserted in a modified version of Addgene vector 160908 (Addgene, Watertown, MA, USA), modified to encode kanamycin resistance instead of hygromycin. This vector contains the RUBY expression cassette for 3 enzymes that synthesize betanin, the red pigment from red beet, previously developed [28]. The complete expression vectors containing both RUBY and the RBD or SPIKE fragments contained 5′UTR before the enhanced 35S promoter with either a SpeI site (ACTAGT) or the 5′-UTR (TACATCACAATCACACAAAACTAACAAAAGATCAAAAGCAAGTTCTTCATGTTGATA) from the *Arabidopsis thaliana* gene encoding alcohol dehydrogenase ADH [29]. This same 5′-UTR from AtADH is present in the GEMINI viral expression vector, which was also tested with the RBD and SPIKE fragment expression (Figure 1), both also containing a C-terminal His Tag.

The Q5 site-directed mutagenesis kit (NEB, Ipswich, MA, USA) was used to include the SpeI and AtADH sequences upstream of the RBD and SPIKE coding sequences in the RUBY vector, following the recommended protocol. The oligonucleotide primers used for mutagenesis are listed in Table 1. The assembled vectors were initially multiplied in *E. coli* Stellar competent HST08 cells (Takara Biosciences, San Jose, CA, USA) after selection on 100 µg/mL spectinomycin and transferred to competent *Agrobacterium tumefaciens* EHA105 for plant transformation and glycerol cryogenic stocks. Whole-plasmid DNA sequencing (Plasmidsaurus, South San Francisco, CA, USA) was used to confirm the absence of unwanted mutations.

### 2.2. Walnut Somatic Embryo Preparation and Plant Transformation

We were the first to develop a transformation protocol for a woody plant like walnut that was based on nut/seed tissues [21]. Nut (zygotic seed) tissues harvested from walnut trees were dissected to remove the zygotic embryo at very early stages in its development, allowing for it to multiply in vitro and to create an immortal repetitive embryogenic culture (REC). A key feature of a REC is that their growth in culture in either a liquid or a solid medium is hormone-autotrophic and that new embryos arise from the surface of the existing embryo. It has been shown that the single epidermal cells (ostensibly embryonic stem cells) distributed over the surface of each embryo undergo division to form new embryos, with the new embryos budding off the surface of existing embryos without any callus (unorganized growth) intermediate [32]. Agrobacterium can transform these epidermal cells as they develop into secondary embryos, and the antibiotic selection process is used to identify the unique and stable transformation events taking advantage of this temporal and spatial nature of RECs [21,33,34]. Using a scorable marker gene like GUS (b-glucuronidase expression), one is able to distinguish between transient and stable events by visualization of GUS gene expression [34]. Additionally, the expression of a scorable marker gene like GUS can be used to identify the high-expressing lines for the gene of interest [35].

J1 paradox (*J. hindsii* × *J. regia* interspecific hybrid) walnut somatic embryos were grown in the dark on DKW medium (PhytoTechnology Laboratories, Inc., Lenexa, KS, USA) at 20–23 °C with weekly transfers to fresh media. *Agrobacterium tumefaciens* (strain EHA105) from glycerol freezer stocks was cultured at 28 °C, 220 rpm in 3 mL of LB selective medium containing 100 µg/mL spectinomycin, 50 µg/mL rifampicin, and 10 µg/mL tetracycline for preparation of the bacterial suspension to be used in plant transformation. After 2 days in culture, 1 mL was transferred to 20 mL of the same medium and grown overnight. OD 600 nm of the bacterial culture was adjusted to 0.5 in a final volume of 20 mL per vector in DKW augmented with 100 µM acetosyringone plus 1 mM proline and incubated for 3 h in preparation for embryo transformation.

Approximately 50 walnut somatic embryo clusters, or ~2 g of total fresh weight, that had recently been transferred to fresh DKW medium to accelerate growth were transferred to the 50 mL tube containing 20 mL of *Agrobacterium* culture to submerge all embryos completely. The tube was then laid horizontally, and transformation occurred at room temperature for 2 h. The bacterial suspension was then discarded, and the embryos were transferred to co-inoculation plates (DKW + 100 µM acetosyringone, 1 mM proline) and maintained at room temperature for 24 h in the dark. The embryos were then washed in 40 mL of DKW + 200 µg/mL of both timentin and kanamycin (selective medium) for 24 h to dilute and suppress the now unwanted viable bacterial cells. The embryos were finally plated on selective medium containing 100 µg/mL of timentin and kanamycin and kept at room temperature in the dark, being transferred to fresh medium weekly for the first two transfers and then every two or three weeks for subsequent transfers. The selection proceeded for 4–6 months, generating independent, non-chimeric, clonal lines corresponding to unique transformation events. This also ensured sufficient clonal embryo material in culture to minimize the risk of losing some to contamination. During this selection period, the cultures were monitored daily, and wild-type embryos that progressively turned dark and failed to propagate in the selective medium were gradually eliminated. Transformed embryos became evident during selection by remaining pearl white in color (or developing intense red coloration if they coexpressed RUBY) and by outgrowing the untransformed material. Another distinctive feature is the formation of new E1 embryos budding off the surface of the E0 embryos used for transformation.

E1 embryos showing good growth were selected, receiving a number for identification; were maintained in culture until E2 embryos had formed; and were established into independent cultures, as originally described in [21]. At this stage, any bacteria used for transformation or wild-type embryogenic tissue had been eliminated from the culture, and the resulting E2 lines were subsequently clonally propagated without selection pressure. This prolonged selection process also helped to eliminate chimeric E2 lines, as ~5% WT cells can persist in transformed tissue that has already been selected for a few months (Sriema Walawage, unpublished results).

### 2.3. Propagation of Selected Embryo Lines and Preparation of Extracts

Defined E2 lines were cultured independently on solid (DKW + 2.2 g/L Gelzan, PhytoTechnology Laboratories, Lenexa, KS, USA) or in liquid DKW medium. The plates were kept at ambient temperature in the dark, while liquid cultures (25 mL) were kept on an orbital shaker at 60 rpm. Biomass accumulation in both systems was determined by taking the fresh weight of embryo clusters, protected in a sterile Petri dish, at every medium transfer. The embryos were blotted on sterile Whatman filter paper and allowed to dry for a few minutes in a laminar flow hood before being transferred to the sterile Petri dish used to tare the balance. Three independent embryo clusters, initially weighing ~300 mg fresh weight each, were monitored for four continuous weeks of growth. The impacts on biomass accumulation were compared between growth conditions, including solid vs. liquid, as well as between wild-type and transgenic embryos.

The preparation of protein extracts from the embryo cultures used ~300 mg of fresh tissue (approximately 4 small embryos) in 1 mL of extraction buffer (0.9% NaCl, 1 mM EDTA, 2 mM Na_2_S_2_O_5_), prepared in round-bottom 2 mL microcentrifuge tubes containing 2 sterile metal beads. Embryo tissue was disrupted in a ball mill TissueLyser (Retsch, Haan, Germany) for 2 min at 30 Hz. The tubes were then centrifuged at 16,000× *g* for 10 min at 4 °C, separating particulate material and cell debris from the supernatant. Five hundred microliters of clarified supernatant was transferred to a new tube, avoiding the lysed cell pellet. The total protein content in each supernatant was determined in triplicates with a Qubit 4 fluorometer (Thermo Fisher Scientific, Waltham, MA, USA).

### 2.4. Detection of Recombinant Proteins

Enzyme-linked immunosorbent assay (ELISA) was used for recombinant protein detection. High-binding EIA flat-bottom 96-well plates (Corning, Corning, NY, USA) were coated overnight at 4 °C with 100 µL of RBD and SPIKE standards diluted 2× serially from 600 to 5 ng/mL for the standard curve, while embryo protein extracts were diluted 1:5 and 1:40 for analysis. Each sample was analyzed in two wells, at least on two separate plates (totaling four measurements or more). The plate was then washed three times with 200 µL per well of 1× PBS + 0.05% tween 20. Blocking was performed with 100 µL of 1× PBS + 1% blocking agent (Bio-Rad Laboratories, Hercules, CA, USA), with three washes performed as before. One hundred microliters per well of 1:1000 diluted anti-HisTag antibody coupled with HRP (Roche Life Science, Indianapolis, IN, USA) was added per well and incubated for one hour before the plate was washed 3× again. TMB substrate (Merck Millipore, Burlington, MA, USA) was added, 100 µL per well, and acidified with the same volume of 1N HCl after 20 min. Absorbance was read at 450 nm on a VersaMax Microplate Reader (Molecular Devices, San Jose, CA, USA), and the average values of two biological replicates with two technical replicates each were fitted in a standard curve built with standard recombinant RBD and Spike (cat. #SCV2-RBD-050P and SCV2-S-050P, eEnzyme, Gaitherburg, MD, USA).

### 2.5. Quantification of Betanin Content from RUBY Expression

Purified betanin standard (Santa Cruz Biotechnology, Dallas, TX, USA) diluted in distilled water was used to build a standard curve, with a stock solution of 50 mg/mL being serially diluted 2× until 1:256. Three technical replicate readings were taken per concentration. The readings were taken at 531 nm, which was experimentally determined to be the maxima by scanning between 530 and 540 nm, the reported absorbance range for betanin. Sample extracts were prepared using ~100 mg fresh embryo tissue from the non-pigmented line GR03, the red pigmented line SA05, and from red beets (*Beta vulgaris* subsp. *vulgaris*) purchased at a local grocery store. Triplicate independent samples from each were prepared in 1 mL of distilled water each and extracted with a TissueLyser as described previously. Following centrifugation, two hundred microliters of clarified supernatant was carefully diluted 5×, and the absorbance of each sample was measured at 531 nm in triplicate using a SmartSpec 3000 spectrophotometer (Bio-Rad Laboratories, Hercules, CA, USA). The values were fitted in the standard curve and adjusted for dilutions to reach measurements of mg of betanin per g of fresh tissue.

The building of the standard curve, determination of extract betanin content, and conversion to mg betanin/g tissue fresh weight were achieved using a custom-built script in the R programming language. A logistic model of absorbance as a function of betanin content was constructed using the base R nonlinear least-squares (nls) function, with the model statement being “Absorbance~L/(1 + exp(−k × (Concentration − x0)))”, where L is the maximum absorbance, k is curve steepness, and x0 is the concentration where the absorbance is L/2. The determination of extract betanin content was performed through rearrangement of the model statement to predict betanin concentration (mg/mL) from the experimentally acquired absorbances. These resulting values were first multiplied by 5 to correct for the previously described 1:5 dilution and then multiplied by the ratio of initial extraction volume (1 mL) to fresh weight in grams to arrive at the betanin content expressed as mg betanin/g fresh weight. The three technical replicates per sample were aggregated and used for the downstream statistical analysis, leaving each tissue type with three biological replicates each. The determination of statistically significant differences in the resulting concentrations was achieved using Dunn’s test, with significant differences between groups determined using a raw *p*-value cutoff of 0.05.

## 3. Results

### 3.1. Validation of DNA Vectors and Selection of Expression Lines

**Figure 2:**
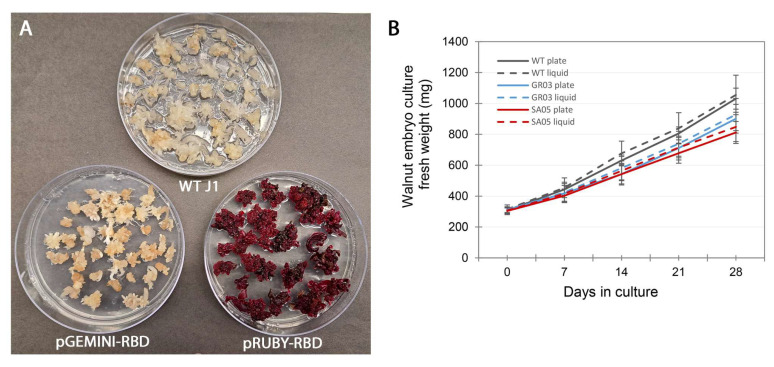
Growth of walnut embryos on solid and liquid media. (**A**) E2 embryo lines growing well on solid medium (in 100 mm Petri dishes) were monitored for biomass increase: pGEMINI-RBD is represented by line GR03, and pRUBY-RBD is represented by line SA05. (**B**) Growth of selected lines observed in liquid DKW medium. Values shown are averages ± standard deviation of three independent flasks.

With the aim of expressing RBD and SPIKE ectodomains in walnut somatic embryos, the construction of expression vectors was confirmed by DNA sequencing, showing all expected components (Figure 1A) and sequence (Figure 1B). Following transformation of the walnut embryos, the selection of transgenic lines proceeded as shown in Table 2. After 4–6 days post-transformation, transient expression of RUBY was observed in many E0 embryos, greatly contrasted with the white wild-type tissue. This initial accumulation of betanin is an indicator of the efficiency of the overall transformation success. The embryos then remained under selection, and after approximately 4 weeks, new tissue clusters with intense red pigmentation emerged. These new E1 somatic embryos, formed from stably transformed single cells, were established as independent E1 lines. The culling of untransformed material through antibiotic selection occurs progressively until the embryos are composed of entirely transformed cells. New embryos stemming from this type of E1 material were established as E2 lines, defined under selection in approximately 3 months, and then cultured independently to accumulate biomass for the analysis of recombinant protein expression and betanin accumulation. Under the strong, enhanced 35S promoter, some lines accumulated enough pigment to appear as dark red as red beets (Figure 2A).

### 3.2. Cultivation and Analysis of Selected Embryo Lines

The E2 lines growing well on selective medium were transferred to individual plates for propagation. Once the wild-type tissues had been eliminated, the embryos approximately doubled in biomass every two weeks (Figure 2B). A total of 57 lines expressing RBD and 34 lines expressing Spike were selected for downstream analysis of recombinant protein expression and pigment production. Considering that ~50 embryos were used in the transformation with each vector, totaling ~350 initial E0 embryos, 95 lines were selected in total using the agroinfection method. As the embryo lines grew, they were grouped for protein extraction into groups of approximately eight to make processing more efficient. The lines GR03, obtained with the Gemini vector, and SA05, obtained with the RUBY vector, were among the fast-growing lines on solid media and were put into liquid culture (Figure 2B). Perhaps due to the metabolic burden of recombinant protein and pigment production, they exhibited a slightly slower growth rate than the wild-type control. On this benchtop scale, the small differences were only noticed at the end of the weekly timepoints upon weighing. Under the test conditions, doubling of biomass occurred in approximately 8–15 days for the wild type, 11–17 days for line GR03 (expressing recombinant RBD), and 13–19 days for line SA05 (expressing recombinant RBD and betanin). Although not statistically significant, the differences observed could widen with longer culture and more observations.

### 3.3. Detection of Recombinant Proteins in Selected Embryo Lines

**Figure 3:**
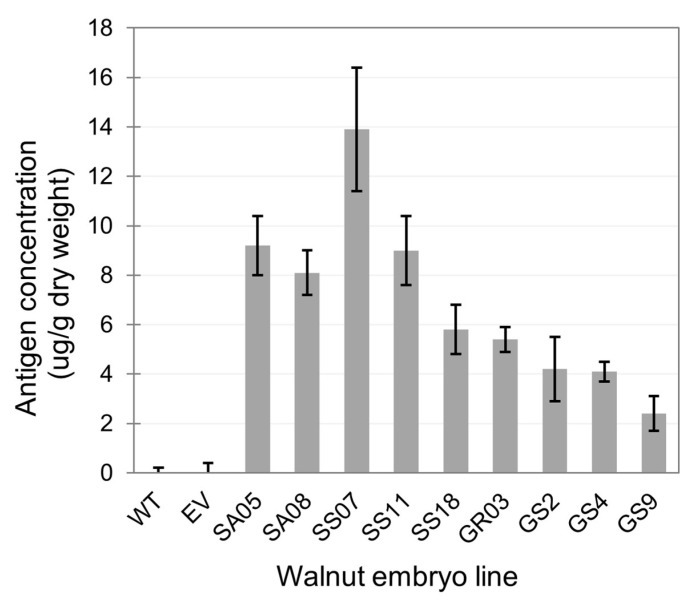
Expression of RBD and SPIKE proteins in selected walnut embryo lines. Quantification of recombinant protein in soluble protein extracts by ELISA using anti-HisTag—HRP—conjugated antibody diluted 1:1000. Values shown are averages ± standard deviation of two independent experiments with two replicates each. Walnut embryo lines with G in the identifier are derived from Gemini vectors, and those with S are derived from RUBY vectors. SA and SS lines express RBD as well as GR, while GS lines express Spike. J1 WT was used as a control for pGEMINI-expressing lines, and pRUBY empty vector (EV) was used as a control for pRUBY-expressing lines.

The selected embryo lines were processed for total protein extraction and quantification of soluble protein. Using the simplified protein extraction procedure (see Methods), the total soluble protein concentrations ranged from ~120 to 650 ng/mL when using 300 mg embryo tissue in 1 mL buffer with the TissueLyser or ~400 to 2200 ng/g of fresh weight tissue. The 1 mL of buffer used with each sample allowed for the recovery of 500 µL or even more of clean supernatant that could be used in several tests. Samples of each extract were assayed for RBD or Spike expression by ELISA, and the best lines from each construct are shown in Figure 3. Most lines had levels of detected recombinant protein in the low ng/mL of fresh extract range, with only some lines achieving higher than 100 ng of recombinant protein/mL of fresh extract. Considering the sample-to-buffer ratio of 300 mg:1 mL buffer and the 95% water content in fresh walnut embryos, the calculated recombinant expression level per mass of dry weight embryo is in the low µg/g range as shown in Figure 3. We initially suspected the titer could be affected by the expression of the RUBY cassette, with the production of the RUBY proteins and betanin metabolite imposing a metabolic burden and restricting the cellular machinery from achieving its maximum target protein production. However, after analyzing many lines from both Gemini and RUBY backgrounds, this appeared not to be the case, as the titers detected in the Gemini-derived extracts were similar to those from RUBY background.

### 3.4. Production of Betanin

**Figure 4:**
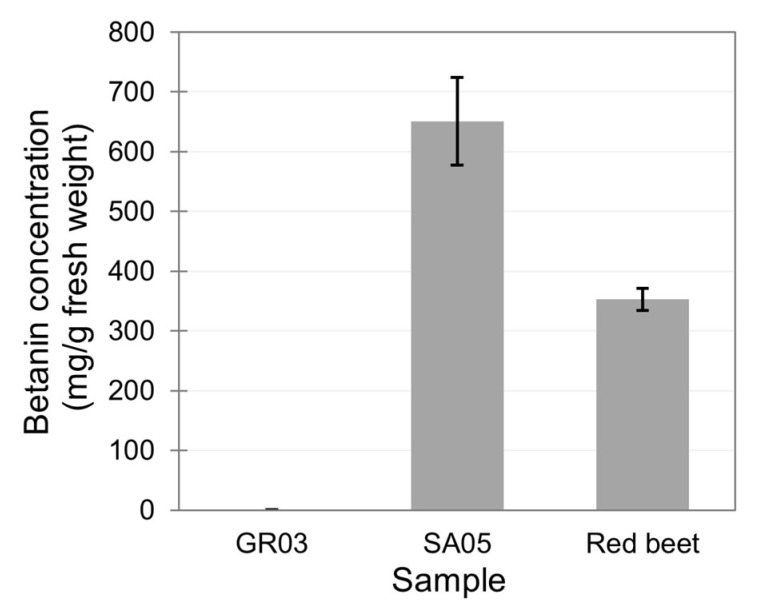
Quantification of betanin produced in walnut somatic embryos. Non-pigmented GR03 walnut embryos were compared with line SA05 expressing RUBY and with an extract prepared from red beet. Quantification was achieved using a calibration curve based on a serially diluted betanin chemical standard, with absorbance measurements taken at 531 nm (within the reported absorbance range for betanin). Spectrophotometric measurements were taken in triplicates from three biological replicates. Average ± standard deviation is shown. Difference between walnut SA05 and red beet considered significant by Dunn’s test (*p*-value < 0.05) and between walnut SA05 and GR03 samples (*p*-value < 0.001).

Embryos transformed with vectors containing the RUBY marker produce and accumulate an intense red pigment. This marker encodes three enzymes that convert tyrosine to betanin. Under a constitutive promoter such as CaMV35S, the red pigment accumulates to high levels, turning the white coloration observed in wild-type embryos into an intense red similar to red beets (Figure 2A). As expected, the spectrophotometric determination of betanin levels in the extracts from the embryo line GR03 not expressing RUBY (obtained with the Gemini vector) did not show any betanin accumulation, while line SA05 expressing RUBY showed a range of 650 ± 73 mg/g of fresh embryo tissue. This level is higher than that detected in red beet fresh tissue (353 ± 19 mg/g fresh tissue) (Figure 4).

## 4. Discussion

Here, we demonstrated the metabolic programmability of walnut somatic embryos for the expression of both recombinant proteins and metabolites. Both RBD and Spike ectodomains were stably expressed in clonally propagated embryo lines without significantly affecting embryo growth. The simultaneous expression of RUBY and consequent accumulation of the red pigment betanin were also detected at very high levels. Recombinantly encoded betanin biosynthesis has been reported in different platforms, including yeast (28.7 mg/L) and rice (170 µg/g dry weight) [36,37]. Notably, the titers obtained in walnut somatic embryos not only surpass these titers by far but also surpass that of red beets, as reported by others and measured by ourselves in this study [38]. To our knowledge, this is the highest level of betanin accumulation reported for any biomanufacturing platform. Despite no absorbance being detected at 531 nm for the white embryos (not expressing RUBY), additional analysis using HPLC to confirm the concentration and absence of other betacyanins would further confirm our preliminary results. In any case, this demonstrates the enormous potential of walnut somatic embryos in producing tyrosine-derived metabolites through metabolic reprogramming. Given the reported phenolic repertoire of walnut, this likely extends beyond tyrosine to include the other derivatives of the shikimic acid pathway.

The ability to produce biomolecules of interest on demand from local sources can alleviate supply chain shortages in times of peak demand while also offering possibilities for novel sources to substitute non-renewables, for example [39,40]. Plant-based platforms for biomanufacturing provide a means to achieve these goals, given that they are (comparatively) inexpensive and can provide high yields in just a few days, such as with transient expression systems in *Nicotiana benthamiana* [41]. Although quick and high-yielding, achieving scaled-up agroinfection requires greater technological investment for efficiency, and expression vector leaf injections are difficult to implement at a large scale. The other option is stable expression, which requires a significantly greater investment of time for the months of selection and establishment of expression lines. This process has room for optimization by increasing single-cell analytical capability and biomass scale-up through cell suspension [42,43].

Among the diverse biomanufacturing platforms, plants are special in their ability to naturally produce a wide range of plastid-derived secondary metabolites. This underlying metabolic framework can be tuned to achieve the biosynthesis of many specialized metabolites with just a couple additional enzymatic steps. This is exemplified through the RUBY-encoded production of betanin, derived from tyrosine, which we demonstrated here in walnut somatic embryos. Another advantage when using plants is the flexibility in expression, both in terms of time and location. In some cases, whole-plant expression of the transgene(s) can quickly be used to achieve very high production levels, such as rice or maize for example [44,45]. Alternatively, specifying expression to particular tissues or during certain developmental stages can be used to co-cultivate food, medicines, and other biomaterials while minimizing footprint and costs. This plasticity is also beneficial in the case of biomolecules that are undesirable for human consumption but desirable to produce. One additional benefit to certain plant systems is that they are, or can produce, edible food. With the walnut embryo being essentially a seed, evidence for safe, direct consumption would be a substantial advantage of the system. This would create the potential for applications in the development of edible vaccines [46,47]. These are stable at room temperature and can be lyophilized for storage and shipping, reducing the costs of distribution and consumption. Finally, one further advantage of the walnut embryo system is that it is naturally devoid of photosynthetic metabolism that generally complicates the purification of metabolites and proteins, particularly if the intended application requires higher purity of the product.

In terms of production of RBD and Spike, the walnut somatic embryo system still requires significant optimization compared to the other benchmarks, like *N. benthamiana*, yeast, fungal C1, and animal CHO cells [48,49,50,51]. These systems can reach titers of g/L after just a few days of fermentation, which can be translated to a productivity of ~100 mg/L/day of recombinant protein. Each production system has its own costs, advantages, and challenges. In this initial study presented here, we obtained recombinant protein titers in the low µg/g dry weight of walnut embryos, much lower than these other expression platforms. Although a direct comparison between a bioreactor system, such as fungal C1 cells, and a continuous growth system, such as the walnut somatic embryos, is difficult, adjusting for the same volume of culture as used for the C1 cells (3.8 L, Ophir et al., in preparation), we can estimate a productivity of ~1.2 µg/L/day for the walnut system. This is at least 1000-fold less than the productivity estimated for the fungal cells. However, this simplified comparison does not consider important factors such as the cost of materials and equipment for each system, which would have to be included in a technical economic analysis. Just as an example, walnut embryos can be cultured at room temperature without light in simple plastic containers. The comparison also must take into account that biomanufacturing in fungal C1 cells is a mature method, already optimized for many years, while the walnut embryo system has not received such investment.

We note a few steps that can be improved if a higher level of product is desired in walnut embryos. To start, a broader study of expression vectors with alternative promoters, terminators, signal peptides, and untranslated regions might yield combinations that give higher expression. Co-expression of stabilizing agents such as chaperones could also be considered. Regarding genetic transformation, without considering other methods, a higher number of walnut embryos can easily be prepared for transformation, potentially rendering more lines to be screened for a higher expression. The random insertion of the T-DNA in the plant genome is a drawback of this method that might be overcome with RNA-guided methods, including CRISPR, or site-specific integrases [52]. Using high-throughput methods that can identify transformed cells for early screening can potentially accelerate the identification of promising lines [53]. Another step that can be optimized is the protein extraction, which can be performed with other extraction buffers and with procedures that render more purified proteins [54]. The simple buffer and procedure used here were for simplicity and compatibility with ELISA. Despite improvements in protein extraction efficiency being possible, organic solvents, strong acid/bases, or any expensive or toxic reagent should be avoided if large-scale production is the goal. Ideally, new biomanufacturing platforms will be more environmentally friendly to better compete with conventional sources.

The growth rate of the embryos can also be optimized. Here, we showed that growth on solid medium is not substantially different from a liquid culture in a rotating flask, but these do not provide optimized aeration. Faster growth can be potentially achieved using intermittent immersion chambers such as RITA^®^ flasks. These could be the inspiration for larger-scale industrial liquid-culture systems. Continuous-flow systems can be engineered so products can be harvested from the growth medium directly [55] or from the growing embryo mass keeping the packed tissue volume. Stirred-tank bioreactors have been used successfully with rice cell suspensions and other plants [18,19]. Walnut cell suspensions can be prepared and be adapted for bioreactors, possibly offering complementary advantages to other species in terms of specialized metabolism such as shikimate derivatives.

One point of concern using tissue culture material for biomanufacturing is contamination, which can be minimized by trained personnel or automation of tissue culture handling. The cost of culture media should also be considered when expanding production. Our study was a small-scale, proof-of-concept demonstration using mg and grams of tissue. To achieve kilogram scale would require alternative containers to Petri plates. Solid media requires, besides the gelling agent that is the highest cost in our culture medium, disposable plastic plates or glassware that needs to be washed. Therefore, liquid-media or whole-plant systems appear necessary for large-scale production of embryogenic material. The DKW medium used here costs approximately $1/L in liquid format. This is comparatively inexpensive for a complex medium, but if novel media can be developed using industry byproducts, for example, further reduction in costs may be possible.

Finally, downstream processing and purification of target molecules are bottlenecks for biomanufacturing on larger scales and can increase production costs significantly. Expression in lipid or protein nanobodies (vesicles) might be useful to purification using simple phase separation (lipid:water) from embryo extracts. Another potential advantage of the walnut system compared to other biomanufacturing benchmarks is safety for direct consumption by animals and maybe even humans, depending on the growth medium, with little or no processing. This might be the factor that compensates for its lower productivity compared to the other platforms, but further research with animal models is still needed.

## 5. Conclusions

Our results show that the tissue culture of walnut somatic embryos is a versatile platform with significant potential for biomanufacturing both proteins and metabolites. Further research is needed to streamline the application on an industrial scale. With the proper technical economic analysis added to the production of biomolecules in high demand, this system holds promise as a source of renewable biomolecules, with the added benefit of being a potentially edible product. With the emergence of new viral variants that require updated vaccine boosts and tests, customized recombinant protein production will continue to be an important part of the response effort, and walnut embryos can now be further studied to complement the supply chain based on other traditional platforms.

## Figures and Tables

**Table 1:** Oligonucleotide primers used for mutagenesis.

Primer ID	Sequence 5′ → 3′
SpeI-RBD-F	TAGTATGAAGAATACCTCTTCCCTTTG
SpeI-R ^1^	GTTTTCAGCGT-GTCCTCTCCAA
SpeI-Spike-F	TAGTATGAAAAACACATCAAGTTTATGCTTATTATTAC
ADH-RBD-F	GATCAAAAGCAAGTTCTTCACTGTTGATAATGAAGAATA-CCTCTTCCCTTTG
ADH-R ^2^	TTTTGTTAGTTTTGTGTGATTGTGATGTATCAGCGTGTCCT-CTCCAA
ADH-Spike-F	GATCAAAAGCAAGTTCTTCACTGTTGATAATGAAAAACA-CATCAAGTTTATGCTTATTATTAC

^1^ The same reverse primer was used with SpeI-Spike-F. ^2^ The same reverse primer was used with ADH-Spike-F.

**Table 2:** Selection of transformed walnut somatic embryos.

Base Vector + 5′UTR Modification	Recombinant Protein	Vector Name	Selected E2 ^1^ Embryo Lines
RUBY + none	None	pRUBY.EV	4
RUBY + SpeI site	RBD	pRUBY.SS	26
RUBY + AtADH	RBD	pRUBY.SA	18
RUBY + SpeI site	SPIKE	pRUBY.LS	8
RUBY + AtADH	SPIKE	pRUBY.LA	15
Gemini + AtADH	RBD	pGEM.RBD	13
Gemini + AtADH	SPIKE	pGEM.SPIKE	11

^1^ Isogenic lines cultured independently for at least 4 months in selective medium.

## Data Availability

The original contributions presented in the study are included in the article, further inquiries can be directed to the corresponding author.
